# Comparative Effectiveness and Safety of Seven Qi-Tonifying Chinese Medicine Injections for AECOPD Patients: A Systematic Review and Network Meta-Analysis

**DOI:** 10.1155/2021/6517515

**Published:** 2021-11-15

**Authors:** Xueyi Deng, Fuqin Kang, Xueyin Chen, Jiaqi Lai, Xuanchen Guan, Xinfeng Guo, Shaonan Liu

**Affiliations:** ^1^The Second Clinical College of Guangzhou University of Chinese Medicine, Guangzhou, China; ^2^The Second Affiliated Hospital of Guangzhou University of Chinese Medicine (Guangdong Provincial Hospital of Chinese Medicine), The Second Clinical College of Guangzhou University of Chinese Medicine, Guangdong Provincial Academy of Chinese Medical Sciences, Guangzhou, China

## Abstract

**Introduction:**

Acute exacerbation of chronic obstructive pulmonary disease (AECOPD) imposes a large burden on economy and society worldwide. In addition to western medicine, multiple kinds of qi-tonifying Chinese medicine injections have been widely used in China as adjunctive treatments. Previous small-sample clinical trials have proven their efficacy in the treatment of AECOPD. However, data on comparative effectiveness and safety of qi-tonifying injections are limited. We conducted this network meta-analysis to compare the efficacy and safety of 7 commonly used qi-tonifying injections in patients with AECOPD.

**Methods:**

Literature search was conducted through electronic databases, including PubMed, the Cochrane Library, EMBASE, CINAHL, AMED, CBM, CNKI, Wanfang database, and VIP database. Randomized clinical trials (RCTs) exploring the efficacy of any of these 7 qi-tonifying injections were included. The primary outcome was lung function (FEV1 and FVC). R 4.0.0 and STATA 12.0 were adopted to perform the network meta-analysis using Bayesian statistics.

**Results:**

A total of 36 RCTs involving 2657 participants were included. The results of network meta-analyses indicated that Chuankezhi injection (CKZ) combined with routine treatment (RT) was superior to other qi-tonifying injections combined with RT in terms of FEV1 improvement (MD = 0.63, 95% CI: 0.22, 1.04). For improving FVC, Shengmai injection (SGM) combined with RT showed the greatest therapeutic effect (MD = 0.38, 95% CI: 0.13, 0.61). Moreover, SGM combined with RT revealed the best estimates for response rate (MD = 4.00, 95% CI: 1.34, 13.63). The main adverse events in this study were gastrointestinal reactions and injection site reactions. No serious adverse events were reported.

**Conclusion:**

In this network meta-analysis, SGM and CKZ were potential best adjunctive therapies in the treatment of AECOPD.

## 1. Introduction

Chronic obstructive pulmonary disease (COPD) is defined by persistent respiratory symptoms and airflow limitation which is due to airway and/or alveolar abnormalities [1]. Over the past few decades, COPD has become a serious public health concern worldwide. COPD caused around 3,000,000 deaths each year globally, making it the 3rd leading cause of deaths [2, 3]. Moreover, due to the increasing environmental exposures (cigarette smoking, ambient particulate matter, etc.) and the aging population [4–7], COPD-related mortality was projected to increase progressively [8]. In China, it was reported that the overall prevalence of COPD was 8.6%, accounting for 99.9 million people [9]; and the death rate was estimated to range from 50 to 100 per 100,000 people [10].

Among them, acute exacerbation of COPD (AECOPD), defined as acute worsening of respiratory symptoms which needs additional therapy [1], is a major factor for the high mortality of COPD. It is established that AECOPD contributes to worse health status, higher rates of readmission, and worse disease progression [11]. Apart from this, the prolonged stay, oxygen therapy, and other medications caused by AECOPD needs made up more than 50% of the total COPD burden on economy and society [12, 13]. Therefore, the treatment of AECOPD is critical for reducing burden of COPD.

The treatment strategies of AECOPD are to minimize the negative impact of the current acute exacerbation and to prevent subsequent events. Currently, routine western medicine for AECOPD mainly includes bronchodilators, corticosteroids, antibiotics, and supplemental oxygen for emergency [1]. Owing to their clinical benefits in relieving symptoms, these therapies are widely used. However, their definite effect in exacerbations remains controversial [14], and their side effects received a growing concern. For instance, corticosteroids are widely applied for the treatment to prevent complications of exacerbation among AECOPD patients. A Danish observational cohort study showed that long course of oral corticosteroids treatment was associated with pneumonia hospitalization or all-cause mortality. Another retrospective cohort study demonstrated that short-term use of oral corticosteroids increased risk of sepsis, venous thromboembolism, and fracture [15, 16]. For the past two decades, multiple studies demonstrated that qi-tonifying Chinese medicine injections, an example of the popular traditional Chinese medicines (TCM) for AECOPD, can overcome acute exacerbation and improve lung function. According to TCM theory, the pathological basis of AECOPD is “exterior excess and interior deficiency.” Qi deficiency runs through the process of AECOPD development, which means qi-tonifying strategy is one of the most important treatment options for AECOPD [17]. Coupled with the high bioavailability of injections [18, 19], multiple qi-tonifying injections were widely used. However, some existing evidence demonstrated that different types of qi-tonifying injections varied in their mechanism of action and clinical efficacy [20–22]. If the best choice of qi-tonifying injections becomes available, clinicians can make better therapeutic choices. However, to date, it is unknown which qi-tonifying injections are more effective; and there were no head-to head studies to compare all these qi-tonifying injections. The Bayesian network meta-analysis can synthesize evidence from direct and indirect comparisons to estimate comparative efficacy [23]. Here, in order to provide the best available treatment, a network meta-analysis was conducted to compare the efficacy and safety of 7 commonly used qi-tonifying injections in patients with AECOPD.

## 2. Methods

The prospective protocol was created and registered in the International Prospective Register of Systematic Reviews (PROSPERO CRD42020200297). The PRISMA checklist for network meta-analysis is presented in Supplementary Materials (Table S1).

### 2.1. Data Sources and Searches

We searched PubMed, the Cochrane Central Register of Controlled Trials, EMBASE, CINAHL Nursing Journal Databases (CINAHL), Allied and Complementary Medicine Database (AMED), Chinese Biomedical Literature Database (CBM), China National Knowledge Infrastructure (CNKI), Wanfang database, and VIP database to find relevant studies from inception to September 20, 2019. All randomized controlled trials (RCTs) in Chinese and English were included without any other restrictions. The search terms and their combinations were “Pulmonary Disease, Chronic Obstructive,” “Randomized controlled trial,” and “Systematic review” combined with seven included injections. These seven included injections were recommended in Chinese medicine monograph and were commonly used in clinical practice [24, 25]. They were as follows: Shenmai injection (SM), Huangqi injection (HQ), Chuankezhi injection (CKZ), Shenqi Fuzheng injection (SQFZ), Shenfu injection (SF), Kangai injection (KA), and Shengmai injection (SGM). Furthermore, the reference lists of the publications were searched for additional articles. The detailed search strategy was described in Supplementary Table S2.

### 2.2. Inclusion Criteria and Exclusion Criteria

We included published RCTs that met the following criteria: (1) trials that enrolled patients with definite diagnostic criteria of AECOPD; (2) trials that explored the efficacy of any of these 7 qi-tonifying injections; (3) qi-tonifying injections were given as intravenous except CKZ, whose conventional usage is intramuscular injection; and (4) trials that reported at least one of the following outcomes: the primary outcome was lung function (including FEV1、FVC); secondary outcomes were FEV1%, arterial blood gas analysis (including PaO_2_ and PaCO_2_), response rate, the six-minute walking distance (6MWD), the length of hospitalization, and modified British medical research council (mMRC). It is noteworthy that response rate was defined according to efficacy criteria [26, 27]. Clinical recovery, markedly effective, effective were classified into response, and noneffective was classified into nonresponse.

Exclusion criteria were as follows: (1) trials that combined other types of Chinese medicine product, such as Chinese medicine decoction, Chinese patent medicine, and acupuncture; (2) duplicate studies; (3) literature review; (4) studies with only abstracts.

### 2.3. Study Selection

All titles and abstracts were screened by two reviewers (Xueyi Deng and Jiaqi Lai), and the full texts of eligible articles were obtained for final inclusion.

### 2.4. Data Extraction and Quality Assessment

Two reviewers (Fuqin Kang and Xuanchen Guan) used a designed form independently to extract and summarize the following data: first author, year of publication, study ID, Journal, study design, sample size, treatment regimens, follow-up time, and adverse event. Two researchers (Xueyi Deng and Jiaqi Lai) independently assessed risk of bias of each study using the Cochrane Risk of Bias Tool [28]. Random sequence generation, allocation concealment, blinding of participants and personnel, blinding of outcome assessment, incomplete outcome data, selective reporting, and other biases were assessed. If any discrepancies were raised, they were resolved by discussion to achieve consensus and arbitration.

### 2.5. Statistical Analysis

For all outcomes, we conducted pairwise meta-analyses in random-effects model using Cochrane collaboration software RevMan (5.3). Odds ratios (ORs) were reported for dichotomous outcomes and mean differences for continuous outcomes. *P* value ＜ 0.05 was considered to be statistically significant. Random-effects network meta-analyses were conducted with STATA (12.0) software and R software (4.0.0) using gemtc package, if there were enough available RCTs for each outcome and intervention. We generated network plots for several outcomes to clarify the direct comparisons or indirect comparisons. The rank probability was generated to show which treatment is the best. Funnel plots and Egger's test were conducted to assess the publication bias.

## 3. Results

### 3.1. Literature Retrieval and Study Characteristics

**Figure 1:**
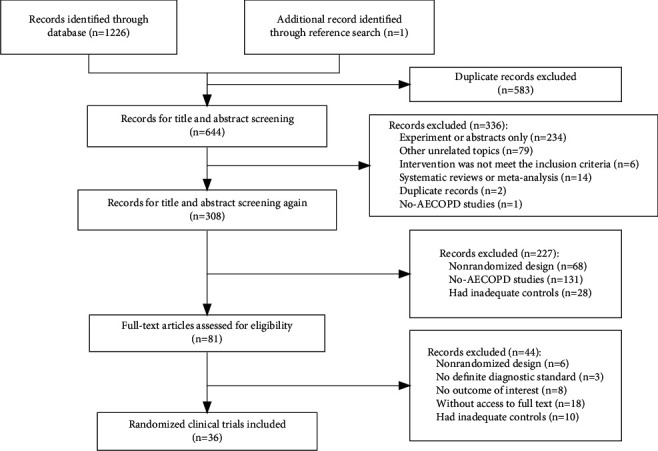
Study flow diagram.

A total of 1226 articles were identified, and 81 articles were assessed for full-text screening. Finally, 36 eligible RCTs involving 2657 participants were included. The details of the literature screening are presented in Figure 1. All 36 studies were published between 2004 and 2019, and the sample size ranged from 36 to 128. There were 31 RCTs reporting the age, and the average age of participants in these studies was 66 years. The characteristics of included studies are reported in Table 1. Overall, baseline characteristics of participants were comparable among different studies. In addition to targeted interventions (7 qi-tonifying injections), all participants received RT, with treatment duration about 2 weeks. The primary outcome lung function (FEV1, FVC) was reported in more than 10 studies.

### 3.2. Risk of Bias Assessment

**Figure 2:**
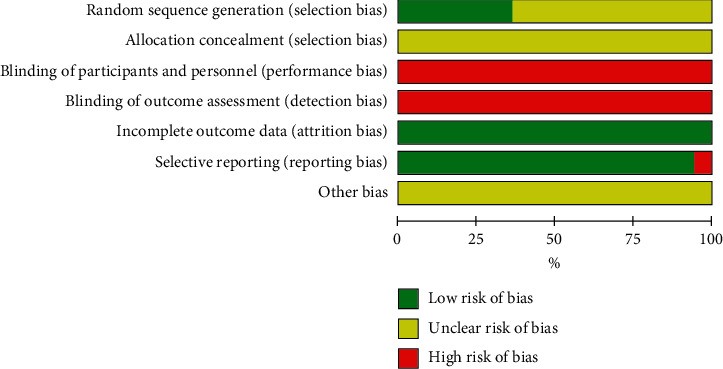
The detailed risk of bias assessments.

For random sequence generation, 13 RCTs [32, 39, 41, 43, 45, 46, 50, 52, 57, 58, 61] performed randomization using the random digital table method or random draws, so they were evaluated as low risk; and the remaining RCTs were assessed as unclear because they only mentioned “random” without providing description of randomization in detail. For allocation concealment, all RCTs were estimated as unclear. 35 studies [29, 37, 39–64] were assessed as high risk in terms of blinding of participants and personnel due to no information on blinding. No studies mentioned blinding of outcome assessors. All included studies were deemed to be low risk on incomplete outcome data. As for selective reporting bias, 2 RCTs [29, 41] were identified as high risk since not all prespecified outcome were reported. For other biases, all RCTs were unclear due to inadequate information. The detailed risk of bias assessments summary is reported in Figure 2.

### 3.3. Results of the Pairwise Meta-Analyses

All included studies were two-arm RCTs. In comparison between 7 qi-tonifying injections combined with RT, respectively, SM (MD = 0.34, 95% CI: 0.22, 0.46), HQ (MD = 0.25, 95% CI: 0.23, 0.27), SQFZ (MD = 0.26, 95% CI: 0.09, 0.42), and SGM (MD = 0.39, 95% CI: 0.29, 0.49) combined with RT showed significant effect in FEV1. In addition, CKZ + RT (MD = 0.62, 95% CI: 0.53, 0.72), SQFZ + RT (MD = 0.20, 95% CI: 0.03, 0.37), and SGM + RT (MD = 0.39, 95% CI: 0.26, 0.51) achieved better FVC compared with RT. Most of the qi-tonifying injections plus RT were superior than RT in arterial blood gases and response rate. Detailed results of pairwise comparisons are summarized in Table S3.

### 3.4. Results of the Network Meta-Analyses

**Figure 3:**
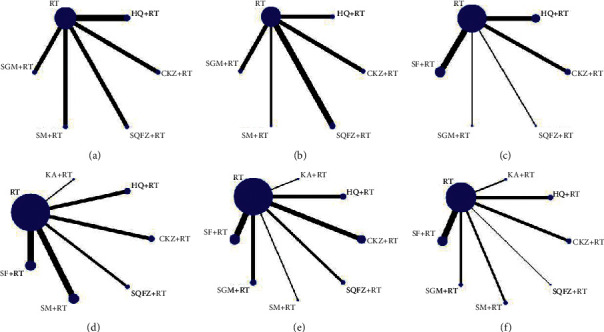
Network meta-analyses of eligible comparisons for lung function (FEV, FVC, and FEV1%), arterial blood gases (PaO_2_ and PaCO_2_), and response rate. (a) FEV1. (b) FVC. (c) FEV1%. (d) PaO_2_. (e) PaCO_2_. (f) Response rate.

**Figure 4:**
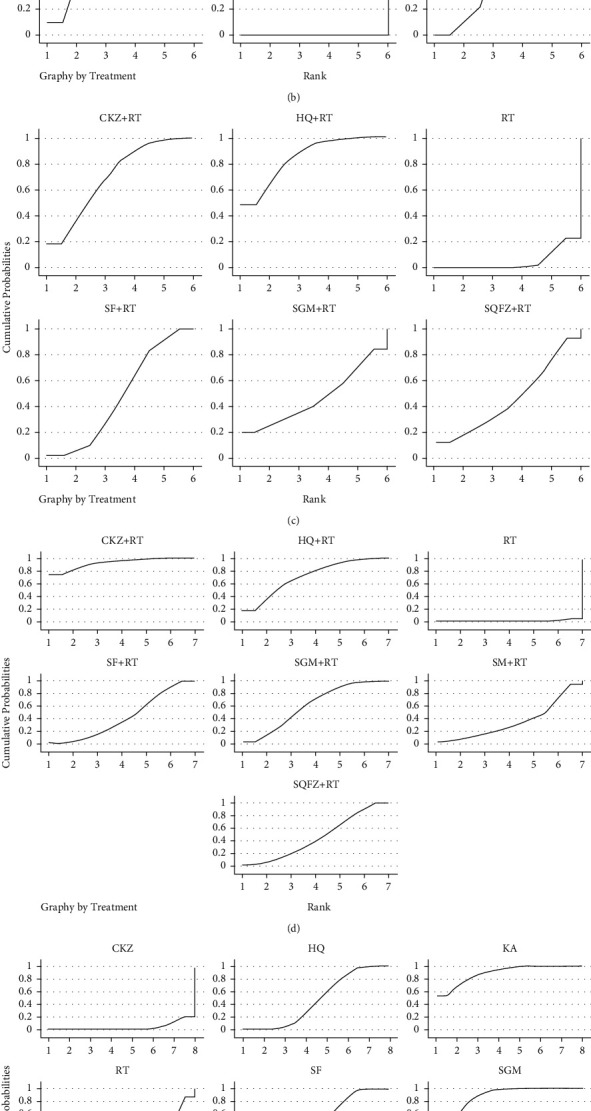
The rank probability of lung function, arterial blood gases (PaO_2_ and PaCO_2_), and response rate for included interventions. (a) FEV1. (b) FVC. (c) FEV1%. (d) PaO_2_. (e) PaCO_2_. (f) Response rate.

For qi-tonifying injections, network meta-analysis included 5 treatments for FEV1 and FVC, 5 treatments for FEV1%, 6 treatments for PaO_2_, and 7 treatments for PaCO_2_ and response rate. The networks of eligible comparisons for lung function, arterial blood gases, and response rate are presented in Figure 3. The pooled estimates of the network meta-analysis are shown in Tables 2–4. The rank probability SUCRA was generated for included interventions and is presented in Figure 4.

#### 3.4.1. Comparison of the Lung Function (FEV1, FVC, and FEV1%)

Of these RCTs, a total of 11 studies [29, 41, 43, 45, 50, 55, 57, 59, 63] reported the outcome of FEV1, involving 5 different qi-tonifying injections. All 5 qi-tonifying injections combined with RT were more beneficial than RT, with MD of 0.38 (95% CI: 0.13, 0.61) for SGM + RT, MD of 0.35 (95% CI: 0.14, 0.58) for SM + RT, MD of 0.25 (95% CI: 0.04, 0.46) for HQ + RT, and MD of 0.26 (95% CI: 0.02, 0.49) for SQFZ + RT. In addition, SGM yielded the best result among these five injections.

In terms of FVC improvement, the random-effects network meta-analyses summarized the MDs for 5 qi-tonifying injections. The results revealed that CKZ combined with RT was associated with the best FVC (MD = 0.63, 95% CI: 0.22, 1.04). Moreover, CKZ + RT was the only treatment that was significantly better than RT, followed by SGM + RT (MD = 0.36, 95% CI: −0.06, 0.78) and SQFZ + RT (MD = 0.21, 95% CI: −0.11, 0.55). Notably, there were no statistically significant differences between SGM + RT, SQFZ + RT, and RT. Therefore, CKZ may be the optimal treatment for improving FVC.

In the analysis of FEV1%, the result of network meta-analyses revealed that HQ + RT may yield the best FEV1% (MD = 6.51, 95% CI: 4.23, 8.85), followed by CKZ (MD = 5.57, 95% CI: 3.14, 8.62) and SF (MD = 4.37, 95% CI: 2.55, 6.51). Both of them combined with RT were approved to be with higher FEV1% when compared with RT alone. There was no significant difference in the association when comparing SGM + RT (MD = 3.65, 95% CI: −4.02,11.45) and SQFZ + RT (MD = 3.70, 95% CI: −2.26, 9.67) with RT alone in network meta-analyses.

#### 3.4.2. Comparison of the Arterial Blood Gases (PaO_2_ and PaCO_2_)

For arterial blood gases, PaO_2_ and PaCO_2_ were reported in 18 RCTs, respectively, in which 6 qi-tonifying injections were evaluated in these trials. The network meta-analyses for PaO_2_ indicated that CKZ (MD = 10.05, 95% CI: 6.02, 15.21) had the highest probability of increasing PaO_2_. Also, HQ + RT (MD = 7.83, 95% CI: 3.57, 11.75), SGM + RT (MD = 6.80, 95% CI: 4.10, 9.98), SQFZ + RT (MD = 5.89, 95% CI: 1.76, 9.83), and SF + RT (MD = 5.68, 95% CI: 2.50, 9.10) were more effective than RT alone.

In terms of PaCO_2_, KA plus RT (MD = −11.71, 95% CI: −19.63, −3.77) was likely to be the best choice. SGM (MD = −10.84, 95% CI: −15.56, −6.05), SQFZ (MD = −8.70, 95% CI: −14.68, −2.79), and SF (MD = −4.22, 95%CI: −7.75 to −0.71) combined with RT resulted in a significantly better outcome than RT. The result of network meta-analysis was consistent with pairwise comparisons. Overall, the effect estimates of SGM were high in both PaO_2_ and PaCO_2_ outcome measurements.

#### 3.4.3. Comparison of the Response Rate

In total, 24 of 36 RCTs [31–40, 42–44, 47–50, 52–54, 56, 58, 60, 62] tested the response rate. For data that were available on all 7 qi-tonifying injections of interest, network meta-analyses were conducted addressing these 7 interventions. SGM + RT (OR = 4.00, 95% CI: 1.34, 13.63) was considered as the best response rate of 7 qi-tonifying injections, although there was no significant difference observed among SGM and the other 6 qi-tonifying injections. Besides, the random-effects network meta-analyses demonstrated that SM + RT (OR = 3.98, 95% CI: 1.57, 11.14), HQ + RT (OR = 3.72, 95% CI: 1.82, 7.87), CKZ + RT (OR = 3.36, 95% CI: 1.56, 7.90), and SF + RT (OR = 2.79, 95% CI: 1.60, 5.05) performed significantly better than RT. However, all of them had similar effects with respect to response rate.

### 3.5. Adverse Events

Ten of the 36 RCTs [30, 32, 35, 40, 42, 47, 53, 54, 60] reported outcomes of adverse events. Among them, 5 RCTs [39, 44, 57, 58, 64] reported no intervention related adverse events, and the other 5 RCTs [30, 32, 41, 42, 47] reported at least one adverse event. A trial evaluated SF reported injection site pruritus (1 case) in the intervention group, and symptom disappeared after withdrawal of infusion [32]. Gastrointestinal reactions were observed in another trial that evaluated SGM, including 4 cases in the treatment group and 3 cases in the control group [47]. Injection-related adverse events (5 cases) were observed after administration of CKZ, including tolerable injection site pain (4 cases) and injection site induration (1 case), which resolved within days after treatment [30]. One patient in CKZ group developed AE symptoms like dizziness and nausea. The symptoms completely disappeared after rest [42]. Serious adverse events were not reported. Further details of side effects are presented in Table 1. Basically, the main adverse events were gastrointestinal reactions and injection site reactions, which would spontaneously relieve without any specific treatment. However, the safety of these seven qi-tonifying injections was still unclear due to limited information.

### 3.6. Publication Bias

**Figure 5:**
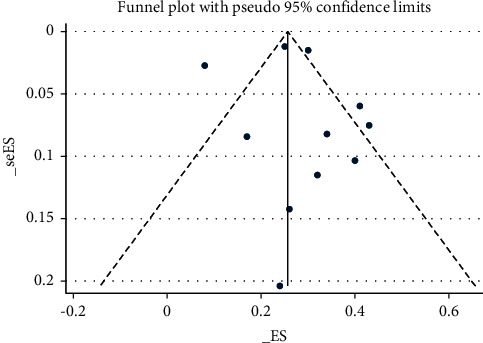
Funnel plot for the publication bias.

Publication bias was evaluated by funnel plot (Figure 5) and Egger's test. There was not any evidence of publication bias for FEV1(*t* = 0.41, *P*=0.691).

## 4. Discussion

In this review, we comprehensively summarized the efficacy and safety of 7 commonly utilized qi-tonifying injections for patients with AECOPD. Our analyses showed that SGM + RT, CKZ + RT, and HQ + RT revealed a highest probability to be the best choice to improve the lung function. In addition, CKZ and KA combined with RT had a similar first ranking in the analysis about arterial blood gases. In terms of response rate, SGM + RT showed the best improvement in network meta-analysis, although no significant difference was observed among these 7 qi-tonifying injections. The wide confidence intervals on ORs may be due to the small sample size. Thus, the results should be treated with caution. In conclusion, our assessment overall found that SGM and CKZ may be the most effective qi-tonifying injections.

Possible explanation about high effect of SGM is that it contains ginsenoside, organic acid, schizandra, and multiple microelements, which may help to decrease pulmonary artery pressure and improve gas exchange function. On the other hand, they can improve hypoxia tolerance by inhibiting Na^+^/K^+^ ATPase to improve myocardial contractility and microcirculation [65, 66]. In addition to the efficacy, the safety of SGM should be considered. However, due to the limited information reported, we cannot draw a specific conclusion. Only 1 RCT reported the side effect related to gastrointestinal reactions [47]. This may be attributed to the excessive secretion of gastric acid and bile promoted by schizandra [67]. Given that, the patient should be evaluated for drug tolerance when SGM was used in excess. Additionally, in 2017, China National Medical Products Administration had informed that SGM-induced allergic shock should be paid more attention in clinical practice [68].

Numerous studies have helped to verify the mechanism of action of epimedins A, B, and C and icariin, which are major constituents of CKZ. It is reported that icariin can not only increase expression of the anti-inflammatory factor interleukin-10 but also decrease expression of various proinflammatory factors IL-8 and tumor necrosis factor-*α*. Besides this, icariin can regulate the expression of Glucocorticoids (GC) resistance-related factors, which was beneficial for reversing GC resistance in COPD [69, 70]. Regarding the administration ways, intravenous mode was the preferred mode of administration (6 studies), followed by intermuscular administration (1 study). Different ways of administration may lead to difference in their bioavailability [19]. In order to get better effect, physicians should adjust the administration strategies timely according to individualized treatment.

### 4.1. Strengths and Limitations

The main strength of this study is that we creatively applied a network meta-analysis to comprehensively compare the efficacy and safety of commonly used qi-tonifying injections. Furthermore, it was not feasible to include all kinds of qi-tonifying injections of interest due to the limited clinical application of some kinds of qi-tonifying injection. Therefore, we focused on seven injections recommended by clinical practice guidelines.

This study had several limitations. First of all, network meta-analyses based on the assumption that comparators among different trials are compared are similar [23, 71]. In addition to characteristics of participants, routine care strategies should be adjusted for any discordance in comparators among trials. However, 25 of 36 trials did not describe routine treatment measures in detail. This may cause inconsistency and heterogeneity. Thus, the limitations need to be considered when interpreting the result. We recommend that the detailed information of RT should be reported in future research. Secondly, response rate was the only outcome being evaluated by all qi-tonifying injections. The bias induced by its subjectivity and uncertainty needs to be noted.

## 5. Conclusions

In conclusion, our results suggested that SGM and CKZ were optimal injections when they combined with RT for the treatment of AECOPD. The safety of these seven qi-tonifying injections was still uncertain due to the limited information. Further studies with direct comparisons of these injections are warranted to confirm our results. Moreover, the safety also needs to be monitored rigorously in the clinical practice.

## Figures and Tables

**Table 1:** Characteristics of included studies.

Study ID	Sample size assessed (I/C)	Mean age (I/C)	Severity	Intervention arm	Control arm	Treatment duration	Reported outcomes	Adverse events (I/C)
Cai et al. [29]	60/60	59.89/61.21	NR	CKZ + RT	RT	2 w	FEV1; PaCO_2_	
Chen et al. [30]	41/43	67.1/65.7	NR	CKZ + RT	RT	14 d	FEV1%	I: tolerable injection site pain (4 cases), injection site induration (1 case)
Chen et al. [31]	55/53	NR	NR	HQ + RT	RT	10～14 d	FVC; response rate	
Chi et al. [32]	48/48	76.45/77.68	NR	SF + RT	RT	14 d	PaO_2_; PaCO_2_; response rate	I: injection site pruritus (1 case)
Deng et al. [33]	30/30	67.5/65.5	NR	SM + RT	RT	10 d	PaO_2_; PaCO_2_; response rate	
Guo et al. [34]	35/35	67/66	NR	SM + RT	RT	15 d	Response rate	
Han et al. [35]	36/36	NR	NR	SF + RT	RT	2 w	FEV1%; PaO_2_; PaCO_2_; response rate	
Hu et al. [36]	43/43	64.39/65.18	NR	CKZ + RT	RT	2 w	FEV1%; 6MWD; response rate	
Zhang et al. [37]	26/25	61/66	NR	HQ + RT	RT	10 d	Response rate	
Jiang et al. [38]	18/18	65.8/66.1	NR	SQFZ + RT	RT	10 d	Response rate	
Jin et al. [39]	34/36	66.44/66.56	NR	SF + RT	RT	2 w	FEV1%; response rate	
Li et al. [40]	36/36	NR	NR	KA + RT	RT	7 d	mMRC; response rate	
Li et al. [41]	42/42	60.3/60.3	1–4	SQFZ + RT	RT	7 d	FEV1; FVC; mMRC	I: oral fungal infection (2 cases), lethargy (1 case), low fever (1 case)
								C: oral fungal infection (1 case), lethargy (1 case)
Li et al. [42]	40/40	60.13/58.81	NR	CKZ + RT	RT	7 d	FEV1; FVC; FEV1%; response rate	I: dizziness, nausea (1 case);
								C: dizziness, nausea (1 case)
Liang et al. [43]	25/25	66.27/65.34	NR	HQ + RT	RT	10 d	FEV1; FEV1%; PaO_2_; PaCO_2_; response rate	
Liao et al. [44]	30/28	68.3/65.2	1–3	SF + RT	RT	14 d	FVC; FEV1%; response rate	
Liu et al. [45]	60/60	65.2/65.0	NR	SQFZ + RT	RT	10 d	FEV1; FVC; FEV1%; PaO_2_; PaCO_2_	
Liu et al. [46]	25/25	68.72/69.56	NR	CKZ + RT	RT	7 d	PaO_2_; PaCO_2_; mMRC	
Lv et al. [47]	36/36	NR	NR	SGM + RT	RT	7 d	PaO_2_; PaCO_2_; response rate	I: gastrointestinal reactions (4 cases);
								C: gastrointestinal reactions (3 cases)
Qin et al. [48]	35/35	60.5/61.3	NR	SF + RT	RT	7 d	FEV1%; PaO_2_; PaCO_2_; response rate	
Ren et al. [49]	35/35	62.5/62.8	NR	SF + RT	RT	2 w	Response rate	
Ruan et al. [50]	64/64	63.4/62.8	2-3	SM + RT	RT	2 w	FEV1; FVC; response rate	
Tang et al. [51]	44/42	72.89/71.23	2–4	SF + RT	RT	7 d	PaO_2_; PaCO_2_	
Wang et al. [52]	30/30	62.8/64.1	NR	SF + RT	RT	NR	PaO_2_; PaCO_2_; the length of hospitalization; response rate	
Wang et al. [53]	32/28	69.5/69.3	1–4	SGM + RT	RT	2 w	PaO_2_; PaCO_2_; the length of hospitalization; response rate	
Wu [54]	25/25	75.35/74	NR	CKZ + RT	RT	7 d	PaO_2_; PaCO_2_; response rate	
Xiao et al. [55]	32/32	63.7/62.6	NR	SM + RT	RT	14 d	FEV1; FEV1%	
Xiong et al. [56]	56/56	66.7/66.5	NR	HQ + RT	RT	14 d	FEV1%; PaO_2_; PaCO_2_; response rate	
Yin et al. [57]	30/30	49.38/47.62	NR	SGM + RT	RT	14 d	FEV1; FVC; FEV1%; PaO_2_; PaCO_2_	
Yuan et al. [58]	39/39	74.4/74.6	1–3	CKZ + RT	RT	21 d	FEV1; FVC; PaO_2_; PaCO_2_; response rate	
Yue et al. [59]	35/35	62.1/61.8	2–3	SGM + RT	RT	2 w	FEV1; FVC; PaO_2_	
Zhang et al. [60]	39/39	64.3/65.1	NR	SF + RT	RT	2 w	FEV1%; PaO_2_; PaCO_2_; response rate	
Zheng et al. [61]	30/28	67.3/67.5	NR	SQFZ + RT	RT	10 d	FVC; PaO_2_; PaCO_2_	
Zhou et al. [62]	31/31	64.63/63.57	NR	SGM + RT	RT	2 w	Response rate	
Zhou et al. [63]	30/30	NR	NR	HQ + RT	RT	14 d	FEV1; FVC; FEV1%	
Zhu et al. [64]	26/26	72.04/71.69	NR	HQ + RT	RT	2 w	FEV1%; PaO_2_; PaCO_2_	

I: intervention; C: control; NR: not reported; CKZ: Chuankezhi injection; HQ: Huangqi injection; SF: Shenfu injection; SM: Shenmai injection; SQFZ: Shenqi Fuzheng injection; SGM: Shengmai injection; KA: Kangai injection; RT: routine treatment.

**Table 2:** Pooled estimates of the network meta-analysis on response rate and FVC.

	Response rate							
FVC	**RT**	**3.36 (1.56, 7.90)**	**3.72 (1.82, 7.87)**	2.85 (0.60, 17.55)	**2.79 (1.60, 5.05)**	**4.00 (1.34, 13.63)**	**3.98 (1.57, 11.14)**	2.62 (0.18, 88.25)
	−**0.63 (**−**1.04,** −**0.22)**	**CKZ** + **RT**	1.10 (0.36, 3.26)	0.86 (0.15, 5.94)	0.83 (0.30, 2.20)	1.20 (0.30, 4.87)	1.19 (0.34, 4.33)	0.78 (0.05, 27.79)
	0.07 (−0.31, 0.51)	**0.70 (0.15, 1.31)**	**HQ** + **RT**	0.76 (0.14, 5.41)	0.75 (0.30, 1.90)	1.07 (0.29, 4.41)	1.07 (0.32, 3.73)	0.70 (0.04, 25.22)
	—	—	—	**KA** + **RT**	0.98 (0.15, 5.26)	1.40 (0.17, 10.23)	1.39 (0.18, 9.25)	0.90 (0.04, 38.29)
	—	—	—	—	**SF** + **RT**	1.43 (0.41, 5.53)	1.43 (0.47, 4.62)	0.94 (0.06, 32.96)
	−0.36 (−0.78, 0.06)	0.27 (−0.31, 0.86)	−0.43 (−1.04, 0.13)	—	—	**SGM** + **RT**	0.99 (0.21, 4.57)	0.65 (0.03, 25.09)
	**0.75 (0.17, 1.33)**	**1.38 (0.67, 2.10)**	0.68 (−0.06, 1.36)	—	—	**1.11 (0.39, 1.83)**	**SM** + **RT**	0.65 (0.04, 24.60)
	−0.21 (−0.55, 0.11)	0.42 (−0.11, 0.95)	−0.28 (−0.84, 0.21)	—	—	0.15 (−0.39, 0.68)	−**0.96 (**−**1.64,** −**0.29)**	**SQFZ** + **RT**

Values in bold indicate statistical difference.

**Table 3:** Pooled estimates of the network meta-analysis on FEV1 and FEV1%.

	FEV1							
FEV1%	**RT**	0.18 (−0.01, 0.45)	**0.25 (0.04, 0.46)**	—	—	**0.38 (0.13, 0.61)**	**0.35 (0.14, 0.58)**	**0.26 (0.02, 0.49)**
	−**5.57 (**−**8.62,** −**3.14)**	**CKZ** + **RT**	0.07 (−0.28, 0.35)	—	—	0.20 (−0.18, 0.49)	0.17 (−0.17, 0.46)	0.07 (−0.29, 0.37)
	−**6.50 (**−**8.85,** −**4.23)**	—	**HQ** + **RT**	—	—	0.13 (−0.20, 0.44)	0.09 (−0.20, 0.41)	0.00 (−0.32, 0.32)
	—	—	—	**KA** + **RT**	—	—	—	—
	**−4.37 (−6.51, −2.55)**	1.21 (−1.98, 4.70)	2.13 (−1.02, 5.05)	−	**SF** + **RT**	—	—	—
	−3.65 (−11.45, 4.02)	1.98 (−6.18, 10.21)	2.86 (−5.25, 10.81)	−	0.74 (−7.21, 8.72)	**SGM** + **RT**	−0.03 (−0.34, 0.31)	−0.12 (−0.45, 0.22)
	—	—	—	—	—	—	**SM** + **RT**	−0.09 (−0.43, 0.22)
	−3.67 (−9.67, 2.26)	1.94 (−4.47, 8.63)	2.83 (−3.56, 9.19)	—	0.71 (−5.50, 7.03)	−0.04 (−9.85, 9.84)	—	**SQFZ** + **RT**

Values in bold indicate statistical difference.

**Table 4:** Pooled estimates of the network meta-analysis on PaO_2_ and PaCO_2_.

	PaO_2_							
PaCO_2_	**RT**	**10.05 (6.02, 15.21)**	**7.83 (3.57, 11.75)**	—	**5.68 (2.50, 9.10)**	**6.80 (4.10, 9.98)**	4.65 (−1.77, 11.10)	**5.89 (1.76, 9.83)**
	−1.76 (−5.80, 2.55)	**CKZ** + **RT**	−2.23 (−9.11, 3.21)	—	−4.40 (−10.38, 0.80)	−3.21 (−9.05, 1.77)	−5.41 (−13.82, 1.92)	−4.15 (−10.98, 1.24)
	4.74 (−0.02, 9.68)	**6.51 (0.05, 12.80)**	**HQ** + **RT**	—	−2.15 (−7.13, 3.38)	−1.00 (−5.67, 4.41)	−3.17 (−10.62, 4.63)	−1.97 (−7.59, 3.84)
	**11.71 (3.77, 19.63)**	**13.48 (4.34, 22.32)**	6.97 (−2.42, 16.21)	**KA** + **RT**	—	—	—	—
	**4.22 (0.71, 7.75)**	**5.98 (0.39, 11.31)**	−0.54 (−6.57, 5.39)	−7.52 (−16.19,1.23)	**SF** + **RT**	1.17 (−3.20, 5.61)	−1.01 (−8.40, 6.04)	0.21 (−5.21, 5.15)
	**10.84 (6.05, 15.56)**	**12.61 (6.10, 18.76)**	6.10 (−0.81, 12.75)	−0.88 (−10.11, 8.38)	**6.62 (0.66, 12.52)**	**SGM** + **RT**	−2.20 (−9.41, 4.70)	−0.90 (−6.24, 3.74)
	3.76 (−4.37, 11.98)	5.52 (−3.73, 14.59)	−1.00 (−10.57, 8.51)	−7.95 (−19.34, 3.43)	−0.45 (−9.37, 8.46)	−7.09 (−16.50, 2.41)	**SM** + **RT**	1.22 (−6.42, 8.68)
	**8.70 (2.79, 14.68)**	**10.46 (3.07, 17.61)**	3.96 (−3.76 11.61)	−3.01 (−12.97, 6.98)	4.49 (−2.44, 11.43)	−2.14 (−9.71, 5.54)	4.94 (−5.17, 15.08)	**SQFZ** + **RT**

Values in bold indicate statistical difference.

## Supplementary Materials

Supplementary Materials S1: PRISMA NMA Checklist of Items to Include When Reporting A Systematic Review Involving a Network Meta-Analysis. S2: search strategy of EMBASE. S3: pairwise random-effects meta-analyses of lung function, arterial blood gases, and response rate.
